# Topographic patterns of white matter hyperintensities are associated with multimodal neuroimaging biomarkers of Alzheimer’s disease

**DOI:** 10.1186/s13195-020-00759-3

**Published:** 2021-01-18

**Authors:** Malo Gaubert, Catharina Lange, Antoine Garnier-Crussard, Theresa Köbe, Salma Bougacha, Julie Gonneaud, Robin de Flores, Clémence Tomadesso, Florence Mézenge, Brigitte Landeau, Vincent de la Sayette, Gaël Chételat, Miranka Wirth

**Affiliations:** 1 German Center for Neurodegenerative Diseases, Dresden, Germany; 2 Department of Child and Adolescent Psychiatry, Psychosomatics and Psychotherapy, LMU University Hospital Munich, Ludwig-Maximilians-Universität, Munich, Germany; 3 Department of Nuclear Medicine, Charité - Universitätsmedizin Berlin, Corporate Member of Freie Universität Berlin, Humboldt-Universität zu Berlin, Berlin Institute of Health, Berlin, Germany; 4 Inserm UMR-S U1237, Caen-Normandie University, GIP Cyceron, Caen, France; 5 Clinical and Research Memory Center of Lyon, Lyon Institute for Elderly, Hospices Civils de Lyon, Lyon, France; 6 Normandy University, UNICAEN, PSL Research University, EPHE, INSERM, U1077, CHU of Caen, Neuropsychology and Imaging of Human Memory, Caen, France

**Keywords:** Cerebrovascular disease, Alzheimer’s disease, Alzheimer’s disease pathology, White matter lesion

## Abstract

**Background:**

White matter hyperintensities (WMH) are frequently found in Alzheimer’s disease (AD). Commonly considered as a marker of cerebrovascular disease, regional WMH may be related to pathological hallmarks of AD, including beta-amyloid (Aβ) plaques and neurodegeneration. The aim of this study was to examine the regional distribution of WMH associated with Aβ burden, glucose hypometabolism, and gray matter volume reduction.

**Methods:**

In a total of 155 participants (IMAP+ cohort) across the cognitive continuum from normal cognition to AD dementia, FLAIR MRI, AV45-PET, FDG-PET, and T1 MRI were acquired. WMH were automatically segmented from FLAIR images. Mean levels of neocortical Aβ deposition (AV45-PET), temporo-parietal glucose metabolism (FDG-PET), and medial-temporal gray matter volume (GMV) were extracted from processed images using established AD meta-signature templates. Associations between AD brain biomarkers and WMH, as assessed in region-of-interest and voxel-wise, were examined, adjusting for age, sex, education, and systolic blood pressure.

**Results:**

There were no significant associations between global Aβ burden and region-specific WMH. Voxel-wise WMH in the splenium of the corpus callosum correlated with greater Aβ deposition at a more liberal threshold. Region- and voxel-based WMH in the posterior corpus callosum, along with parietal, occipital, and frontal areas, were associated with lower temporo-parietal glucose metabolism. Similarly, lower medial-temporal GMV correlated with WMH in the posterior corpus callosum in addition to parietal, occipital, and fontal areas.

**Conclusions:**

This study demonstrates that local white matter damage is correlated with multimodal brain biomarkers of AD. Our results highlight modality-specific topographic patterns of WMH, which converged in the posterior white matter. Overall, these cross-sectional findings corroborate associations of regional WMH with AD-typical Aß deposition and neurodegeneration.

## Introduction

White matter hyperintensities (WMH), as quantified using fluid-attenuated inversion recovery (FLAIR) magnetic resonance imaging (MRI), are recognized as a marker of cerebrovascular disease [1] that is robustly associated with age [2, 3] as well as cardiovascular risk factors [4, 5]. The presence of WMH is also related to lower cognitive performance [6, 7] and increased risk of clinical Alzheimer’s disease (AD) ([8, 9], for review see [10]), suggesting an involvement of WM damage in AD pathogenesis.

Previous studies have suggested that WM pathology could be directly related to pathological hallmarks of beta-amyloid (Aβ) plaques, tau tangles, and neurodegeneration, measured using sensitive in vivo positron emission tomography (PET), cerebrospinal fluid (CSF), or magnetic resonance imaging (MRI) biomarkers. Thus, greater presence and extend of WMH were previously associated with greater Aβ deposition in some cross-sectional studies [11–15], although not consistently found [16], as well as lower glucose metabolism [17] and reduced gray matter volume (GMV) [2] in brain regions typically affected by AD. At the same time, tau pathology was not consistently related to global (whole brain) or regional WMH, with some authors reporting effects [18–20], not found by others [11, 12].

In the existing literature, global WMH descriptors are often used to quantify WM pathology and to assess respective relationships with brain biomarkers of AD. This methodological approach is masking out specific regional effects. Therefore, voxel-wise mapping methods were recently used to determine topographic patterns of WM lesions that are associated with Aβ and/or tau burden [11, 12]. Findings across these independent cohort studies provide convergent support for the involvement of periventricular posterior brain regions in the association between Aβ deposition and regional WMH, which is not seen for tau burden. Similarly, a recent study demonstrated an age-dependent increase in posterior WM alterations (as assessed by mean diffusivity and WMH) in individuals with abnormal Aβ deposition across the life span [21].

The present study aimed to extend previous findings and corroborate a possible link between multimodal brainbiomarkers of AD and regional WM damage. Here, we systematically investigated associations of spatial WMH distribution, as determined using regions-of-interest (ROIs) and the voxel level approach, with neocortical Aβ burden, temporo-parietal hypometabolism, and medial-temporal GMV reduction. All neuroimaging biomarkers were assessed in the same participants selected to span the broad continuum of cognitive abilities from cognitively normal to mild cognitive impairment and AD dementia. Our main objective was to highlight topographic patterns of WM lesions that are associated with AD-typical Aβ deposition and neurodegeneration.

## Material and methods

### Participants

All participants took part in a larger multimodal imaging study of early-stage AD (“Imagerie Multimodale de la maladie d’Alzheimer à un stade Précoce”, IMAP+) conducted in Caen (France). The subset of cognitively normal participants was included in our earlier study on WMH in IMAP+ [3]. Inclusion and exclusion criteria of the IMAP+ cohort are detailed in our previous publications [3, 22, 23]. Briefly, participants were all aged ≥ 50 years, had at least 7 years of education, and had no history of alcoholism, drug abuse, head trauma, or psychiatric disorders. The IMAP+ study was approved by the regional ethics committee (Comité de Protection des Personnes Nord-Ouest III) and registered at http://clinicaltrials.gov (no. NCT01638949). All participants gave their written informed consent to the study prior to enrollment.

Participants were assigned to groups of either older controls (OC) or individuals with subjective cognitive decline (SCD), mild cognitive impairment (MCI), or AD dementia. Diagnostic criteria for all diagnostic groups are described in a previous publication [22, 23]. In brief, OC were recruited from the general population through advertisement or word of mouth. Participants with SCD, MCI, and AD were recruited from local memory clinics and selected according to internationally agreed criteria (see below). Clinical diagnoses were assigned by a multidisciplinary panel of senior neurologists and neuropsychologists.

The OC and SCD participants had cognitive performances in the normal age-related range on a standardized neuropsychological examination. The presence of SCD was diagnosed, when cognitive concerns were self-reported to the clinician during the interview and on a cognitive complaint questionnaire, as previously described [24, 25]. Patients with MCI were diagnosed according to the criteria of single or multiple domain amnestic MCI [26, 27], concluding memory complaints, objective episodic memory deficits, normal general cognitive status, normal daily functioning, and absence of dementia. Patients with AD were diagnosed using standard clinical criteria of the National Institute of Neurological and Communicative Disorders and Stroke and the Alzheimer’s Disease and Related Disorders Association (NINCDS-ADRDA) for probable AD [28].

Participants were selected from the IMAP+ database, when both a high-resolution FLAIR and a T1 scan were available. For PET measurements, no acquisition or bad image quality resulted in the absence of AV45-PET scans for 6 OC, 2 participants with SCD, and 2 MCI and 2 AD patients and the absence of FDG-PET scans for 1 OC, 1 participant with SCD, and 1 MCI and 1 AD patient (Table 1). All MRI and PET assessments were performed in close temporal proximity (within 3 months).

**Table 1:** Sample characteristics

Variable	All	OC	SCD	MCI	AD	***p***
	***n*** = 155	***n*** = 51	***n*** = 28	***n*** = 51	***n*** = 25	
Age, years	69.9 (7.8)	70.8 (6.4)	67.1 (7.4)	73.3 (7.2)	68 (10.2)	.002^a,d,f^
Sex, *n* females (%)	86 (55.5)	24 (47.1)	17 (60.7)	29 (56.9)	16 (64)	.466
Education, years	11.9 (3.7)	12.2 (3.8)	13.4 (3.5)	11 (3.6)	11.6 (3.3)	.045^d^
ApoE, allele 4 carriers (%)^#^	49 (33.6)	10 (19.6)	3 (11.1)	21 (44.7)	15 (71.4)	< .001^b,c,d,e,f^
MMSE, /30^#^	26.7 (3.8)	28.7 (1.2)	29 (1.1)	26.7 (1.9)	20 (4.5)	< .001^b,c,d,e,f^
Systolic blood pressure, mmHg^#^	142.4 (21.5)	145.2 (20.8)	135.3 (22.6)	145 (23.4)	138.4 (16.2)	.171
Diastolic blood pressure, mmHg^#^	80.1 (12.4)	82.6 (12.4)	78.5 (12.4)	79.1 (13.3)	78.7 (10.2)	.401
AV45-PET SUVR in AD meta-signature^#^	1.28 (.25)	1.13 (.13)	1.14 (.14)	1.36 (.25)	1.53 (.25)	< .001^b,c,d,e,f^
FDG-PET SUVR in AD meta-signature^#^	1.38 (.13)	1.45 (.08)	1.47 (.1)	1.34 (.09)	1.24 (.16)	< .001^b,c,d,e,f^
GMV in AD meta-signature, mL	7.81 (1.03)	8.29 (.68)	8.31 (.68)	7.32 (1.07)	7.24 (1.15)	< .001^b,c,d,e^
WMH volume, mL	10.14 (11.24)	6.34 (6.9)	8.21 (9.65)	12.98 (15.07)	14.27 (13.52)	.005^b,c,e^

*OC* older controls, *SCD* participants with subjective cognitive decline, *MCI* patients with mild cognitive impairment, *AD* patients with Alzheimer’s disease, *ApoE* apolipoprotein E, *MMSE* Mini-Mental State Examination, *AV45* florbetapir, *FDG* fluorodeoxyglucose, *GMV* gray matter volume, *WMH* white matter hyperintensities, *mmHg* millimeter of mercury, *mL* milliliter

Results are presented as mean (standard deviation) for continuous variables. Percentage of females and percentage of allele 4 carriers are also reported for sex and ApoE, respectively. Significant differences at *p* < .05 between ^a^OC and SCD, ^b^OC and MCI, ^c^OC and AD, ^d^SCD and MCI, ^e^SCD and AD, and ^f^MCI and AD. ^#^Missing data for OC/SCD/MCI/AD participants: ApoE, 0/1/4/4; MMSE, 0/0/0/1; blood pressure measures, 3/4/1/0; AV45-PET, 7/2/2/2; FDG-PET, 1/1/1/1

### MRI acquisition and processing

All MRI scans were acquired on a 3-T Achieva scanner (Philips, The Netherlands). Participants first underwent high-resolution T1-weighted anatomical volume imaging using a 3D fast field echo (FFE) sequence (3D-T1-FFE sagittal; repetition time = 20 ms, echo time = 4.6 ms, flip angle = 20°, 180 slices, slice thickness = 1 mm, no gap, field of view = 256 × 256 mm^2^, matrix = 256 × 256, in-plane resolution = 1 × 1 mm^2^). This acquisition was followed by a FLAIR acquisition using a 3D-IR sagittal sequence (repetition time = 8000 ms, echo time = 348 ms, flip angle = 90°, 90 slices with no gap, slice thickness = 2 mm, field of view = 250 × 250 mm^2^, matrix = 320 × 320, in-plane resolution = .78 × .78 mm^2^).

Structural MRI data were segmented and warped to Montreal Neurologic Institute (MNI) template space using the “Segment” procedure implemented in voxel-based morphometry (VBM) [29, 30] using multichannel images (T1 and FLAIR) and Statistical Parametric Mapping 12 (SPM) software (Wellcome Trust Centre for Neuroimaging, London, UK) to obtain maps of gray matter (GM). After binarizing gray matter maps in template space with a threshold of .5, total GMV was extracted using modality-specific AD meta-signature regions, as previously established in an independent cohort [31]. The gray matter meta-signature regions that comprised regions in the bilateral medial-temporal cortices found to show reduced GMV in an independent sample of AD patients from the Alzheimer’s Disease Neuroimaging Initiative (ADNI) database. Moreover, total intracranial volume (TIV) was computed as the sum of volumes of GM, WM, and CSF using the SPM “Tissue volume” routine.

Processing of FLAIR scans is described in detail in our previous publication [3]. Briefly, WMH probability maps were computed using lesion prediction algorithm (LPA) as implemented in the lesion segmentation toolbox (LST) (version 2; https://www.applied-statistics.de/lst.html) for SPM (www.statistical-modelling.de/lst.html) [32] based on T1 and FLAIR images. Processing included a registration of FLAIR images to respective T1 scans and computation of native-space lesion probability maps. To quantify regional WMH volumes, native-space lesion probability maps were binarized (threshold = .5) with a minimal cluster extent of 10 mm^3^. Next, two processing pipelines were applied to evaluate the distribution of WMH (see below).

### ROI-based processing

Total WMH volume was extracted from different regions-of-interest (ROIs) in cerebral WM. ROIs were selected in accordance with the previously published STRIVE criteria [1]. In total, the following twelve ROIs were created: the four lobes of the brain (WM in frontal, temporal, parietal, and occipital), four major WM tracts (corona radiata, external capsule, internal capsule, and optic radiation), and three subsections of the corpus callosum (WM of genu, body, and splenium), as reported by an earlier study [12]. These ROI masks were calculated by aggregating different WM regions from the Desikan atlas [33] for lobes and corpus callosum or the ICBM-DTI-81 WM labels atlas [34] for WM tracts. In addition, a global cerebral WM mask was created including the whole cortex, excluding ventricles, brainstem, and cerebellum. Binary ROI masks were computed in MNI template space and projected back onto individual native-space images using the inverse transformation matrices calculated during the VBM procedure. Total WMH volumes were calculated for each ROI and divided by TIV (described below) to take into account the variability of brain volumes.

### Voxel-based processing

Native-space WMH probability maps were warped to MNI space using deformation fields previously computed by VBM processing. Then, warped probability maps were smoothed using an isotropic 6-mm full-width at half-maximum (FWHM) kernel. A WMH frequency map across the whole cohort was computed by averaging individual warped WMH probability maps from all participants binarized at *p* > .5 and masked by the global cortical mask (described above).

### PET acquisition and processing

Participants also underwent AV45-PET and FDG-PET scanning within 3 months after MRI. Data was acquired on a Discovery RX VCT 64 PET-CT scanner (General Electric Healthcare, USA) with a resolution of 3.76 × 3.76 × 4.9 mm (field of view = 157 mm). Forty-seven planes were obtained with a voxel size of 1.95 × 1.95 × 3.27 mm. A transmission scan was performed for attenuation correction prior to PET acquisition. For AV45-PET acquisition, ≈ 4 MBq/kg of AV45 was injected intravenously to each subject. Then, a 20-min PET scan, beginning 50 min after the injection, was acquired. For FDG-PET acquisition, participants were fasted for at least 6 h before tracer injection. After a 30-min resting period in a quiet and dark environment, ≈ 180 MBq of FDG was intravenously injected as a bolus. The PET acquisition scan began 50 min post-injection for a duration of 10 min.

Both AV45- and FDG-PET data were co-registered to the corresponding T1-weighted MRI image and warped to MNI space using the deformation fields calculated during the VBM procedure (described above). PET images were then semi-quantitatively scaled using whole cerebellar and brainstem pons as a reference, respectively, for AV45- and FDG-PET [32] to obtain standardized uptake value ratio (SUVR) images.

Mean SUVR values were extracted within brain regions typically affected in AD, using modality-specific AD meta-signature regions [31]. The AD meta-signature for AV45-PET included all neocortical regions, excluding para-hippocampi, pre- and post-central gyri, and occipital cortices. The FDG-PET meta-signature included temporo-parietal, precuneal, and posterior cingulate cortices of both hemispheres.

### Assessment of additional measures

The Mini-Mental State Examination (MMSE) [35] was administrated to all participants to measure global cognitive functioning. Education was measured by years of formal schooling. Systolic blood pressure (SPB) and diastolic blood pressure (DBP) were assessed either before the MRI or/and PET scans (in the latter case, an average value was computed). Blood pressure measures were unavailable for eight subjects and were thus replaced by the mean of all SBP measures over all available participants. Genotyping of apolipoprotein E (APOE) was established in accordance with standard procedures: restriction of isotyping from genomic DNA extracted from frozen leukocytes, amplification by PCR, and restriction with *Hha*I [36]. Based on this procedure, all participants were classified as ApoE ε4 carrier when at least one allele of ε4 was present.

### Statistical analyses

Statistical analyses were performed using IBM SPSS Statistics, version 23.0 (IBM Corp., Armonk, NY). Sample characteristics were evaluated as follows. One-way analysis of variance (ANOVA) models with group (OC, SCD, MCI, or AD) as fixed factor was used for continuous variables and Student’s *t* test for pairwise differences. Between-group differences for categorical variables were assessed using the Freeman–Halton extension of Fisher’s exact probability test and *χ*^2^ (chi-squared) tests for pairwise comparison. The distribution of continuous biomarkers measured at the regional level was assessed for approximate symmetry using visual inspection of Q–Q plots. Global and regional TIV-adjusted WMH volumes and mean AV45-SUVR values were thereafter log-transformed to reduce skewness and conform measures more closely to normal distribution. Statistical analyses were conducted across the entire cognitive spectrum, in order to assess a range of individuals in whom there is sufficient variance in brain pathology due to Aβ deposition, neurodegeneration, and WM damage.

ROI analyses were performed in the entire sample using partial correlation analyses to assess the relationship between total log-transformed WMH volumes in each ROI and each of the AD brain biomarkers (mean AV45-SUVR, mean FDG-SUVR, and total GMV in modality-specific AD meta-signature regions). Age, sex, education, and SBP were added as covariates of non-interest. Results are presented at *p* < .05 uncorrected. In addition, correction for multiple comparisons using Bonferroni correction (corresponds to *p* < .05/12 = .0042) is presented.

Voxel-based analyses were performed in the entire sample using SPM12. Three models of multiple regressions were computed with warped and smoothed probability maps of WMH as dependent factor; mean AV45-SUVR (log-transformed), mean FDG-SUVR, and total GMV in AD meta-signature regions (one at a time) as independent variables; and the same covariates of non-interest as for ROI analyses (i.e., age, sex, education and SBP, and TIV for the last model). Results are presented at *p* < .005 uncorrected at peak level with a cluster extend of > 150 voxels. In addition, a more conservative threshold at *p* < .005 uncorrected at peak level in combination with a correction for multiple comparisons (family-wise error [FWE] correction) at the cluster level *p* < .05 was applied.

## Results

### Sample characteristics

Demographic, clinical, and biomarker characteristics of the sample are presented in Table 1. A total of 155 participants, 51 OC, 28 SCD, 51 MCI, and 25 AD patients, were included in this study. Across diagnostic groups, a significant effect of age (*p* = .002) and a minor effect of years of education (*p* = .045) were found. Groups were comparable in gender distribution as well as SBP or DBP (all *p*’s > .1). MMSE scores, number of ApoE ε4 carriers, AD neuroimaging biomarkers, and total WMH volumes significantly differed across groups (all *p*’s < .01).

### Analyses in regions-of-interest

Results of the ROI-based analyses are presented in Table 2. There were no significant associations between mean cortical AV45-SUVR values and WMH volumes in any of the twelve ROIs (all *p*’s > .1 corrected and uncorrected). Lower mean FDG-SUVR was significantly associated with greater WMH volume, mainly in the whole cerebrum, the posterior lobes, corona radiata, internal capsule, and body and splenium of the corpus callosum (all *p*’s < .05 corrected). Lower GMV was significantly related to greater WMH volume in all ROIs (all *p*’s < .05 corrected). In addition, both neurodegeneration biomarkers were related to the optic radiation at the more liberal uncorrected threshold. No significant positive associations were observed for FDG-SUVR and GMV biomarkers.

**Table 2:** Relationships of AV45-SUVR, FDG-SUVR, and GMV with WMH volume in regions-of-interest

Regional WMH volume		AV45-SUVR		FDG-SUVR		GMV	
		***r***	***p***	***r***	***p***	***r***	***p***
i.	Global WM	.056	.517	− .300	< .001^a,b^	− .354	< .001^a,b^
	Frontal WM	− .030	.724	− .035	.677	− .372	< .001^a,b^
	Temporal WM	.002	.977	− .153	.064	− .396	< .001^a,b^
	Occipital WM	.064	.453	− .300	< .001^a,b^	− .409	< .001^a,b^
	Parietal WM	.045	.602	− .242	.003^a,b^	− .380	< .001^a,b^
ii.	Corona radiata	.026	.763	− .285	< .001^a,b^	− .382	< .001^a,b^
	External capsule	.060	.485	− .097	.245	− .319	< .001^a,b^
	Internal capsule	.056	.513	− .371	< .001^a,b^	− .421	< .001^a,b^
	Optic radiation	.039	.647	− .198	.016^a^	− .175	.032^a^
iii.	Genu corpus callosum	.035	.684	− .164	.047^a^	− .312	< .001^a,b^
	Body corpus callosum	.114	.184	− .256	.002^a,b^	− .247	.002^a,b^
	Splenium corpus callosum	.130	.128	− .443	< .001^a,b^	− .442	< .001^a,b^

*SUVR* standardized uptake value ratio, *AV45* florbetapir, *FDG* fluorodeoxyglucose, *GMV* gray matter volume, *TIV* total intracranial volume, *WM* white matter

Results of the Pearson correlation analysis, adjusted for covariates of age, sex, education, and systolic blood pressure. WMH volumes in regions-of-interest (ROIs) were adjusted for TIV and log-transformed; AV45-SUVR were also log-transformed. ROI in (i) brain lobes, (ii) WM tracts, and (iii) corpus callosum. ^a^Significant result at *p* < .05 uncorrected. ^b^Significant result at *p* < .05 Bonferroni-corrected

### Voxel-based analyses

**Fig. 1:**
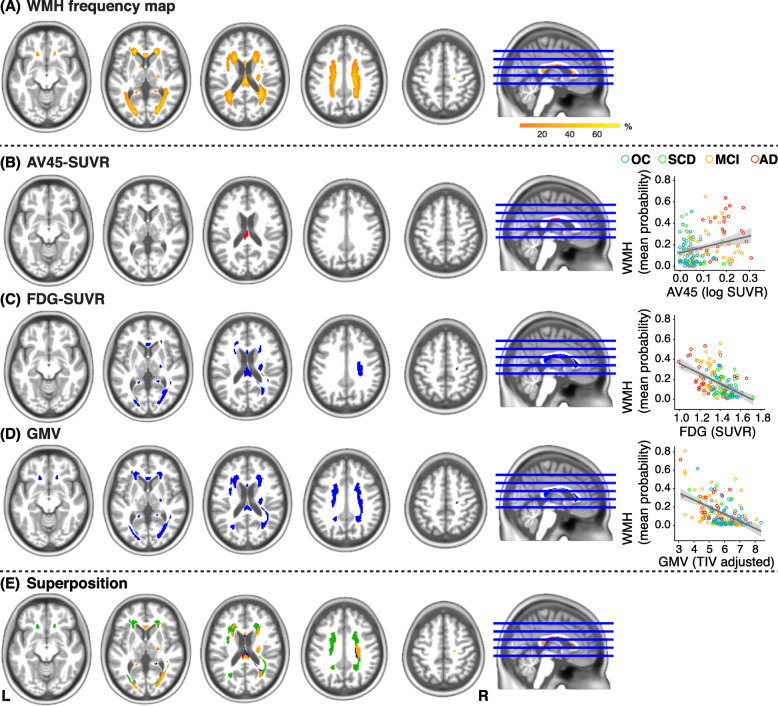
Frequency map and results of voxel-based analyses. **a** WMH frequency map across the whole cohort (*n* = 155) thresholded at 5% used as an explicit mask in regressions analyses. **b**–**d** Relationships between AV45-SUVR (**b**), FDG-SUVR (**c**), GMV (**d**), and spatial WMH distribution at the voxel level (dependent variable). Topographic patterns are displayed at *p* < .005 uncorrected. Corresponding scatter plots visualize the respective relationships using unadjusted values for the whole cohort. Circles represent individual data points (blue = OC, green = SCD, orange = MCI, red = AD), lines indicate linear trends, gray-shaded areas indicate 95% confidence intervals. **e** Superposition of the three topographic patterns (**b**, **c**, and **d**) with blue = FDG-SUVR only, green = GMV only, orange = superposition FDG-SUVR/GMV, and red = superposition AV45-SUVR/FDG-SUVR/GMV. *OC* older controls, *SCD* participants with subjective cognitive decline, *MCI* patients with mild cognitive impairment, *AD* patients with Alzheimer’s disease, WMH white matter hyperintensities, SUVR standardized uptake value ratio, AV45 florbetapir, FDG fluorodeoxyglucose, GMV gray matter volume, TIV total intracranial volume.

The frequency map of WMH in our whole cohort is presented in Fig. 1a. All lobar regions comprised WMH, with more than half of the participants presenting WMH in frontal and parietal mainly periventricular regions.

Results of voxel-wise analyses are presented in Table 3 and Fig. 1b–d. There was no significant positive association between the global AV45-SUVR and regional WMH distribution (*p* < .005 uncorrected, *p* < .05 FWE cluster-level correction). At a more liberal statistical threshold (*p* < .005 uncorrected), WMH in the splenium extending to the body of the corpus callosum were associated with greater AV45-SUVR. No significant negative associations were found between WMH and AV45-SUVR at the given statistical thresholds. Regional WMH were negatively associated with temporo-parietal FDG-SUVR. This pattern included posterior WM (parietal and occipital lobes), the splenium of the corpus callosum extending to the body and genu of the corpus callosum and the right corticospinal tract (*p* < .005 uncorrected, *p* < .05 FWE cluster-level correction). Medial-temporal GMV was also negatively associated with regional WMH in many brain regions, including posterior regions, the corpus callosum, and the superior and anterior corona radiata, but also in the optic radiation and the corticospinal tract. No significant positive associations were observed for FDG-SUVR and GMV biomarkers.

**Table 3:** Relationships of AV45-SUVR, FDG-SUVR, and GMV with WMH at voxel level

Regression WMH with	Label	Hemisphere	Cluster		Peak of cluster			
			***p***	Size	***z*** value	MNI coordinates		
						***x***	***y***	***z***
*AV45-SUVR*	Splenium corpus callosum^a^	Left	.392	199	3.45	− 6	− 32	18
*FDG-SUVR*	Splenium corpus callosum^a,b^	Left	.004	2321	7.37	− 4	− 33	20
	Corticospinal tract^a,b^	Right	.01	1803	6.09	30	− 18	33
	Inferior occipital^a^	Left	.205	361	4.28	− 20	− 87	2
	Superior frontal-occipital fasciculus^a^	Right	.255	288	3.33	24	14	22
	Superior frontal-occipital fasciculus^a^	Left	.173	419	3.07	− 22	3	22
*GMV*	Body corpus callosum^a^	Both	.044	1031	7.68	0	27	4
	Anterior corona radiata^a,b^	Right	< .001	6449	6.89	15	30	− 10
	Anterior corona radiata^a,b^	Left	< .001	4264	6.54	− 18	33	− 8

*SUVR* standardized uptake value ratio, *AV45* florbetapir, *FDG* fluorodeoxyglucose, *GMV* gray matter volume, *MNI* Montreal Neurologic Institute, *TIV* total intracranial volume

Results of linear regression analysis between AV45-SUVR, FDG-SUVR, and GMV and the smoothed WMH probability maps, adjusted for age, sex, education, systolic blood pressure, and TIV (only for GMV). Anatomical labels, coordinates in MNI template space, and *z* values of the cluster peak are provided as well as cluster *p* value and cluster size. ^a^Significant result at *p* < .005 uncorrected. ^b^Significant result at *p* < .005 uncorrected with family-wise error correction (*p* < .05) at the cluster level

Superposition of the three topographic patterns (Fig. 1e), i.e., associations of AV45-SUVR, FDG-SUVR, and GMV with voxel-based WMH (Fig. 1b–d), converged on a common region in the posterior part of the corpus callosum (Fig. 1e, in red). Moreover, most of the WM regions associated with FDG-SUVR overlapped with the GMV-associated WM pattern (Fig. 1e, in orange) predominately in posterior regions.

In a *post hoc* sensitivity check, we restricted our statistical analyses (ROI-based and voxel-based) to non-demented participants, excluding participants with AD. Adjusting for age, sex, education, systolic blood pressure, and TIV (for GMV), significant associations between regional WM alterations and GMV as well as FDG-SUVR were maintained, albeit at a less stringent *p* value threshold. We did not find a significant correlation between regionalWMH and AV45-SUVR, likely reflecting lower presence of AD pathology in the restricted sample.

## Discussion

This study examined associations between regional WMH, as assessed in regions-of-interest and voxel-wise, with multimodal brain biomarkers of AD across a broad cognitive spectrum. WMH in periventricular frontal and parietal areas were mainly associated with temporo-parietal hypometabolism as well as medial-temporal atrophy. While topographic patterns varied across imaging modalities, WM alteration in the posterior white matter was associated with greater manifestation of both neurodegeneration and Aβ pathology (albeit at a more liberal threshold). Together, these cross-sectional findings corroborate associations between regional WM damage and brain biomarkers of AD pathophysiology.

### Association between WMH and Aβ deposition

There was no significant relationship between neocortical Aβ burden and region-specific WMH. Voxel-wise WMH correlated with Aβ deposition at a more liberal statistical level in a small posterior cluster localized to the splenium of the corpus callosum. Interestingly, this specific topographic pattern was also found in previous reports, using PET or CSF measures of Aβ pathology [12, 37]. Other imaging studies with cognitively normal or non-demented older participants have shown associations between regional WMH (frontal and parietal) and elevated Aβ deposition at baseline and over time [11, 38]. By contrast, more global descriptorsof WM pathology appeared to be unrelated to Aβ burden in cognitively normal older participants [39, 40], while a significant relationship was seen in a mixed cohort of patients with cognitive impairment [13].

In the present study, the association between regional WMH and AD-typical Aβ deposition was subtle and limited in statistical power. When restricting our analyses to non-demented participants, the relationship was no longer significant. This could be explained by our moderate sample size, the lower range of AD pathology in the non-demented subgroup, or by the global quantification of Aβ deposition using AD meta-signature regions [31]. To re-evaluate the respective relationship, future studies could be enriched by patients with biological evidence of AD through inclusion of AD biomarkers in diagnostic criteria. In addition, existing variations across studies may be explained by differences in the manifestation of vascular conditions and other pathologies in the enrolled participants.

### Association between WMH and neurodegeneration

We found that WMH, including regions in the posterior lobes, the corpus callosum, and anterior areas, were associated with brain biomarkers of AD-typical neurodegeneration. In accordance with our findings, global WMH burden was previously associated with metabolic and structural patterns of brain injury commonly seen in AD [2, 17, 41]. In more detail, topographic WMH patterns that correlated with temporo-parietal hypometabolism and medial-temporal GMV reduction incorporated posterior lobes (parietal, occipital), parts of the corpus callosum (mostly splenium and body), and the corona radiata. Additional associations were found in bilateral frontal WM regions and WM tracts, such as external capsule, though more prominently seen for the GMV biomarker.

The present neurodegeneration biomarkers are sensitive to AD pathology [42, 43]; however, they are not specific. Nonetheless, the distribution of associated WM damage, particularly within posterior brain regions, converges with the topographic pattern detected in a previous study using CSF biomarkers of AD pathology [12]. Notably, WMH and other vascular factors were previously associated with lower glucose metabolism in frontal regions [44–46]. We report a similar relationship with temporo-parietal glucose metabolism in this and our earlier study [17]. The latter association was identified by our specific methodological approach, i.e., correlating glucose metabolism as measured in AD signature regions (namely temporo-parietal, precuneal and posterior cingulate cortex) with the spatial WMH distribution. Discrepancies in the regional manifestation of metabolic dysfunction, associated with WMH, could be explained by the presence of mixed etiologies underlying WMH (vascular or AD-related pathology) and need to be further investigated.

### Synopsis

Overall, this cross-sectional study confirmed associations between regional WMH distribution and multimodal brain biomarkers of AD. Interestingly, topographic patterns of the observed WMH effects converged in the splenium of the corpus callosum, mirroring previous findings in clinical samples. In AD patients, significantly greater WMH were found in posterior periventricular regions and the splenium of the corpus callosum compared to healthy controls, with MCI patients in the intermediate range [47]. On the other hand, WM atrophy in the posterior corpus callosum was formerly linked to lower posterior glucose metabolism in AD patients, a relationship that was independent of WMH [48]. Together, the evidence appears to support an association between AD pathophysiology and posterior WM alteration, which is suggested to play a role in cognitive dysfunctions seen in AD [47].

We cannot specify the sequence of pathophysiological events underlying our observations. It is possible that AD pathology disrupts regional WM integrity or, vice versa, WM injury of presumed vascular origin hastens AD pathogenesis. For example, it was proposed that posterior WM damage may occur in parallel with or secondary to AD neurodegenerative alterations through a loss of intra-cortical neural connections [48, 49]. Deposition of Aβ may be associated with cerebral amyloid angiopathy, a small vessel disease frequently seen in AD patients (for review see, [50]), thought to contribute to regional WM damage. On the other hand, it is also possible that a vascular pathogenic event, such as ischemia, may induce both WM injury and degenerative pathology or increase the vulnerability of the brain to AD pathogenesis.

### Strengths and limitations

Strengths of our study encompass the inclusion of multimodal neuroimaging biomarkers of AD to systematically investigate associated topographic patterns of WMH within regions-of-interest and at the voxel level. Also, the present monocentric cohort incorporated the cognitive continuum from cognitive normality to mild cognitive impairment and AD dementia. All neuroimaging data were acquired in the same participants using the same MRI and PET scanners and underwent standardized quality control. Such methodical characteristics are of advantage for, e.g., automatic segmentation algorithms. We believe that the unique combination of high-quality multimodal neuroimaging biomarkers outwaits to some extend the moderate sample size and complements previous studies using a similar methodological approach [11, 12].

Limitations of our study include the cross-sectional nature of the design, which precludes to draw direct conclusions on causality. Larger longitudinal studies will be needed to clarify temporal sequences of pathological events, underlying neurophysiological mechanisms, and disease stage-dependent specificities. Further, we defined AD dementia using standard clinical diagnostic criteria, not including biological markers of AD [51]. Some of the enrolled participants may not develop AD dementia, which, in turn, may affect the specificity of our findings. We included the automated lesion segmentation tool LPA to segment FLAIR images. Although this algorithm is reliable and valid [52], performance of automated segmentation methods is imperfect comprising under- and overestimations. False positive detections are often reported in the corticospinal tract; thus, results in this tract should be considered carefully. Our statistical models were adjusted for systolic blood pressure, in accordance with a previous study [11]. Additional vascular risk factors and other cerebrovascular lesions, such as cerebral lacunes or microbleeds, may also play a role in the evaluated associations and should be considered in prospective studies. Even though age was included as a covariate, it is likely that not all effects of age on the neuroimaging biomarkers were fully accounted for. This is especially important for the neurodegeneration biomarkers, even when derived within AD-typical brain regions. There was a substantial percentage of relatively young participants in our AD group. Post hoc exploratory analyses did not reveal any substantial differences between AD patients below and above 65 years of age, considering all imaging biomarkers including WMH sub-regions. However, due to limited statistical power, we cannot entirely preclude an influence of age of onset in the AD group. While regional WMH measures were inspected for normality and log-transformed, a similar procedure was not implemented at the voxel level. Given the exploratory nature of our analyses, we also did not apply more conservative non-parametric methods. It is recommended that biomarker associations reported in our study are further evaluated in follow-up studies including neuroimaging data of larger samples and non-parametric techniques. Finally, it would be worthwhile to re-assess the topographic association between WMH and tau accumulation, which was not accessible for the existing sample.

## Conclusion

The present study supports an association between AD-typical Aβ deposition and neurodegeneration with topographic patterns of WMH. Our findings further highlight that regional WMH associated with the AD brain biomarkers converged in posterior regions. Longitudinal studies are necessary to establish the role of regional WM damage in the pathophysiological sequence of AD.

## Data Availability

The datasets used and analyzed during the current study are available from the corresponding author on reasonable request.

## References

[1] Wardlaw JM, Smith EE, Biessels GJ, Cordonnier C, Fazekas F, Frayne R, Lindley RI, O'Brien JT, Barkhof F, Benavente OR (2013). Neuroimaging standards for research into small vessel disease and its contribution to ageing and neurodegeneration. Lancet Neurol.

[2] Habes M, Erus G, Toledo JB, Zhang T, Bryan N, Launer LJ, Rosseel Y, Janowitz D, Doshi J, Van der Auwera S (2016). White matter hyperintensities and imaging patterns of brain ageing in the general population. Brain.

[3] Garnier-Crussard A, Bougacha S, Wirth M, Andre C, Delarue M, Landeau B, Mezenge F, Kuhn E, Gonneaud J, Chocat A (2020). White matter hyperintensities across the adult lifespan: relation to age, Abeta load, and cognition. Alzheimers Res Ther..

[4] de Leeuw FE, de Groot JC, Oudkerk M, Witteman JC, Hofman A, van Gijn J, Breteler MM (2002). Hypertension and cerebral white matter lesions in a prospective cohort study. Brain.

[5] Cox SR, Lyall DM, Ritchie SJ, Bastin ME, Harris MA, Buchanan CR, Fawns-Ritchie C, Barbu MC, de Nooij L, Reus LM (2019). Associations between vascular risk factors and brain MRI indices in UK Biobank. Eur Heart J.

[6] Benson G, Hildebrandt A, Lange C, Schwarz C, Kobe T, Sommer W, Floel A, Wirth M (2018). Functional connectivity in cognitive control networks mitigates the impact of white matter lesions in the elderly. Alzheimers Res Ther.

[7] Kloppenborg RP, Nederkoorn PJ, Geerlings MI, van den Berg E (2014). Presence and progression of white matter hyperintensities and cognition: a meta-analysis. Neurology.

[8] Debette S, Schilling S, Duperron MG, Larsson SC, Markus HS (2019). Clinical significance of magnetic resonance imaging markers of vascular brain injury: a systematic review and meta-analysis. JAMA Neurol.

[9] Mortamais M, Artero S, Ritchie K (2014). White matter hyperintensities as early and independent predictors of Alzheimer’s disease risk. J Alzheimers Dis.

[10] Prins ND, Scheltens P (2015). White matter hyperintensities, cognitive impairment and dementia: an update. Nat Rev Neurol.

[11] Graff-Radford J, Arenaza-Urquijo EM, Knopman DS, Schwarz CG, Brown RD, Rabinstein AA, Gunter JL, Senjem ML, Przybelski SA, Lesnick T, Ward C, Mielke MM, Lowe VJ, Petersen RC, Kremers WK, Kantarci K, Jack CR, Vemuri P (2019). White matter hyperintensities: relationship to amyloid and tau burden. Brain..

[12] Weaver NA, Doeven T, Barkhof F, Biesbroek JM, Groeneveld ON, Kuijf HJ, Prins ND, Scheltens P, Teunissen CE, van der Flier WM, Biessels GJ (2019). Cerebral amyloid burden is associated with white matter hyperintensity location in specific posterior white matter regions. Neurobiol Aging.

[13] Yi HA, Won KS, Chang HW, Kim HW (2018). Association between white matter lesions and cerebral Abeta burden. PLoS One.

[14] Zhou Y, Yu F, Duong TQ (2015). White matter lesion load is associated with resting state functional MRI activity and amyloid PET but not FDG in mild cognitive impairment and early Alzheimer’s disease patients. J Magn Reson Imaging.

[15] Brickman AM, Guzman VA, Gonzalez-Castellon M, Razlighi Q, Gu Y, Narkhede A, Janicki S, Ichise M, Stern Y, Manly JJ (2015). Cerebral autoregulation, beta amyloid, and white matter hyperintensities are interrelated. Neurosci Lett.

[16] Roseborough A, Ramirez J, Black SE, Edwards JD (2017). Associations between amyloid β and white matter hyperintensities: a systematic review. Alzheimers Dement.

[17] Wirth M, Villeneuve S, Haase CM, Madison CM, Oh H, Landau SM, Rabinovici GD, Jagust WJ (2013). Associations between Alzheimer disease biomarkers, neurodegeneration, and cognition in cognitively normal older people. JAMA Neurol.

[18] McAleese KE, Firbank M, Dey M, Colloby SJ, Walker L, Johnson M, Beverley JR, Taylor JP, Thomas AJ, O'Brien JT, Attems J (2015). Cortical tau load is associated with white matter hyperintensities. Acta Neuropathol Commun.

[19] Tosto G, Zimmerman ME, Hamilton JL, Carmichael OT, Brickman AM (2015). The effect of white matter hyperintensities on neurodegeneration in mild cognitive impairment. Alzheimers Dement.

[20] Erten-Lyons D, Woltjer R, Kaye J, Mattek N, Dodge HH, Green S, Tran H, Howieson DB, Wild K, Silbert LC (2013). Neuropathologic basis of white matter hyperintensity accumulation with advanced age. Neurology.

[21] Caballero MÁ, Song AZ, Rubinski A, Duering M, Dichgans M, Park DC, Ewers M (2020). Age-dependent amyloid deposition is associated with white matter alterations in cognitively normal adults during the adult life span. Alzheimers Dement..

[22] Mutlu J, Landeau B, Gaubert M, de La Sayette V, Desgranges B, Chételat G (2017). Distinct influence of specific versus global connectivity on the different Alzheimer’s disease biomarkers. Brain.

[23] Wirth M, Bejanin A, La Joie R, Arenaza-Urquijo EM, Gonneaud J, Landeau B, Perrotin A, Mezenge F, de La Sayette V, Desgranges B, Chetelat G (2018). Regional patterns of gray matter volume, hypometabolism, and beta-amyloid in groups at risk of Alzheimer’s disease. Neurobiol Aging.

[24] Perrotin A, La Joie R, de La Sayette V, Barré L, Mézenge F, Mutlu J, Guilloteau D, Egret S, Eustache F, Chételat G (2017). Subjective cognitive decline in cognitively normal elders from the community or from a memory clinic: differential affective and imaging correlates. Alzheimers Dement.

[25] Kuhn E, Moulinet I, Perrotin A, La Joie R, Landeau B, Tomadesso C, Bejanin A, Sherif S, De La Sayette V, Desgranges B (2019). Cross-sectional and longitudinal characterization of SCD patients recruited from the community versus from a memory clinic: subjective cognitive decline, psychoaffective factors, cognitive performances, and atrophy progression over time. Alzheimers Res Ther.

[26] Petersen RC, Morris JC (2005). Mild cognitive impairment as a clinical entity and treatment target. Arch Neurol.

[27] Petersen RC, Negash S (2008). Mild cognitive impairment: an overview. CNS Spectr.

[28] McKhann G, Drachman D, Folstein M, Katzman R, Price D, Stadlan EM (1984). Clinical diagnosis of Alzheimer’s disease: report of the NINCDS-ADRDA Work Group under the auspices of Department of Health and Human Services Task Force on Alzheimer’s Disease. Neurology.

[29] Ashburner J, Friston KJ (2005). Unified segmentation. Neuroimage.

[30] Ashburner J, Friston KJ (2000). Voxel-based morphometry--the methods. Neuroimage.

[31] Besson FL, La Joie R, Doeuvre L, Gaubert M, Mezenge F, Egret S, Landeau B, Barre L, Abbas A, Ibazizene M (2015). Cognitive and brain profiles associated with current neuroimaging biomarkers of preclinical Alzheimer’s disease. J Neurosci.

[32] Schmidt P. Bayesian inference for structured additive regression models for large-scale problems with applications to medical imaging. PhD thesis: Ludwig-Maximilians-Universität München; 2017.

[33] Desikan RS, Segonne F, Fischl B, Quinn BT, Dickerson BC, Blacker D, Buckner RL, Dale AM, Maguire RP, Hyman BT (2006). An automated labeling system for subdividing the human cerebral cortex on MRI scans into gyral based regions of interest. NeuroImage.

[34] Hua K, Zhang J, Wakana S, Jiang H, Li X, Reich DS, Calabresi PA, Pekar JJ, van Zijl PC, Mori S (2008). Tract probability maps in stereotaxic spaces: analyses of white matter anatomy and tract-specific quantification. Neuroimage.

[35] Folstein M, Folstein S, McHugh P (1975). “Mini-mental state”: a practical method for grading the cognitive state of patients for the clinicians. J Psychiatr Res.

[36] Hixson JE, Vernier DT (1990). Restriction isotyping of human apolipoprotein E by gene amplification and cleavage with HhaI. J Lipid Res.

[37] Chao LL, Decarli C, Kriger S, Truran D, Zhang Y, Laxamana J, Villeneuve S, Jagust WJ, Sanossian N, Mack WJ (2013). Associations between white matter hyperintensities and β amyloid on integrity of projection, association, and limbic fiber tracts measured with diffusion tensor MRI. PLoS One.

[38] Moscoso A, Rey-Bretal D, Silva-Rodríguez J, Aldrey JM, Cortés J, Pías-Peleteiro J, Ruibal Á, Aguiar P (2020). White matter hyperintensities are associated with subthreshold amyloid accumulation. NeuroImage.

[39] Marchant NL, Reed BR, Sanossian N, Madison CM, Kriger S, Dhada R, Mack WJ, Decarli C, Weiner MW, Mungas DM, Chui HC, Jagust WJ (2013). The aging brain and cognition: contribution of vascular injury and abeta to mild cognitive dysfunction. JAMA Neurol..

[40] Hedden T, Van Dijk KRA, Shire EH, Sperling RA, Johnson KA, Buckner RL (2012). Failure to modulate attentional control in advanced aging linked to white matter pathology. Cereb Cortex.

[41] Appel J, Potter E, Bhatia N, Shen Q, Zhao W, Greig MT, Raj A, Barker WW, Potter H, Schofield E (2009). Association of white matter hyperintensity measurements on brain MR imaging with cognitive status, medial temporal atrophy, and cardiovascular risk factors. AJNR Am J Neuroradiol.

[42] Hanseeuw BJ, Betensky RA, Schultz AP, Papp KV, Mormino EC, Sepulcre J, Bark JS, Cosio DM, LaPoint M, Chhatwal JP (2017). Fluorodeoxyglucose metabolism associated with tau-amyloid interaction predicts memory decline. Ann Neurol.

[43] Maass A, Landau S, Baker SL, Horng A, Lockhart SN, La Joie R, Rabinovici GD, Jagust WJ (2017). Comparison of multiple tau-PET measures as biomarkers in aging and Alzheimer’s disease. Neuroimage.

[44] Kuczynski B, Jagust W, Chui HC, Reed B (2009). An inverse association of cardiovascular risk and frontal lobe glucose metabolism. Neurology.

[45] Tullberg M, Fletcher E, DeCarli C, Mungas D, Reed BR, Harvey DJ, Weiner MW, Chui HC, Jagust WJ (2004). White matter lesions impair frontal lobe function regardless of their location. Neurology.

[46] Haight TJ, Landau SM, Carmichael O, Schwarz C, DeCarli C, Jagust WJ (2013). Dissociable effects of Alzheimer disease and white matter hyperintensities on brain metabolism. JAMA Neurol.

[47] Yoshita M, Fletcher E, Harvey D, Ortega M, Martinez O, Mungas DM, Reed BR, DeCarli CS (2006). Extent and distribution of white matter hyperintensities in normal aging, MCI, and AD. Neurology.

[48] Teipel SJ, Hampel H, Pietrini P, Alexander GE, Horwitz B, Daley E, Möller HJ, Schapiro MB, Rapoport SI (1999). Region-specific corpus callosum atrophy correlates with the regional pattern of cortical glucose metabolism in Alzheimer disease. Arch Neurol.

[49] McAleese KE, Walker L, Graham S, Moya ELJ, Johnson M, Erskine D, Colloby SJ, Dey M, Martin-Ruiz C, Taylor JP (2017). Parietal white matter lesions in Alzheimer’s disease are associated with cortical neurodegenerative pathology, but not with small vessel disease. Acta Neuropathol.

[50] Iadecola C (2013). The pathobiology of vascular dementia. Neuron.

[51] Jack CR, Bennett DA, Blennow K, Carrillo MC, Dunn B, Haeberlein SB, Holtzman DM, Jagust W, Jessen F, Karlawish J (2018). NIA-AA research framework: toward a biological definition of Alzheimer’s disease. Alzheimers Dement.

[52] Heinen R, Steenwijk MD, Barkhof F, Biesbroek JM, van der Flier WM, Kuijf HJ, Prins ND, Vrenken H, Biessels GJ, de Bresser J (2019). Performance of five automated white matter hyperintensity segmentation methods in a multicenter dataset. Sci Rep.

